# Exploring the acceptability of a decision aid for rural women with a history of prior cesarean birth regarding subsequent mode of birth in Coatepeque, Guatemala

**DOI:** 10.3389/fgwh.2024.1261040

**Published:** 2024-10-30

**Authors:** Andrea Jimenez-Zambrano, Morgan Avery, Kathryn Feller, Claudia Rivera, Angela Marchin, Antonio Guillermo Bolaños, Edwin Asturias, Hector Rodas, Margo S. Harrison

**Affiliations:** ^1^ Department of Pediatrics, University of Colorado, Aurora, CO, United States; ^2^ Colorado School of Public Health, Center for Global Health, Aurora, CO, United States; ^3^ Physician Assistant Program, University of Colorado, Aurora, CO, United States; ^4^ Department of Obstetrics & Gynecology, University of Colorado, Aurora, CO, United States; ^5^ Center for Human Development, Ritalhuleu, Guatemala; ^6^ Department of Obstetrics and Gynecology, Coatepeque Hospital, Coatepeque, Guatemala

**Keywords:** beliefs, attitudes, decision aid, mode of birth after cesarean birth, TOLAC counseling

## Abstract

**Background:**

Decisions regarding mode of delivery in the context of a prior cesarean birth is complicated because both trial of labor after cesarean and elective repeat cesarean birth have risks and benefits.

**Purpose:**

The objective of this study was to understand the perspective of women and obstetricians in Coatepeque, Guatemala, to guide the development of a decision aid about mode of birth for women with a history of prior cesarean.

**Methods:**

We conducted in-depth semi-structured interviews with obstetricians at Coatepeque Hospital and women at the Center for Human Development in the southwest Trifinio region of Guatemala in February 2020. Using qualitative content analysis, we recorded, transcribed, translated, and analyzed qualitative data for the meaning of themes and concepts exploring the acceptability of counseling with a decision aid regarding mode of delivery.

**Results:**

A total of 30 qualitative interviews were conducted with women and physicians. Three themes emerged from the qualitative interviews: Having a decision aid for women with a prior cesarean birth will be useful and helpful. Content of the decision aid should include benefits and risks for women and babies as well as figures. Women described the need of tailoring the content surrounding family's role in their decisions. They felt that a trusted provider from the healthcare system should facilitate the use of the decision aid for counseling.

**Conclusions:**

These findings emphasize the support and need for innovative approaches to patient education around mode of delivery after a prior cesarean in the southwest region in Guatemala. There is a need to improve the educational information given to women regarding their mode of delivery after a cesarean birth. Finally, an effective decision aid needs to be tailored to not only the women's needs but also the engagement of the family unit for its successful implementation.

## Introduction

Latin America has the highest cesarean birth rates in the world (1). Rates have risen in Guatemala from 16%–26%, over the past decade (2, 3). From 2015–2017 cesarean birth rates increased from 30%–45% in the southwest Trifinio region (1–3). Once a woman has a scarred uterus from a cesarean, she might be able deliver by elective repeat cesarean or attempt a trial of labor (vaginal birth) (4). As cesarean birth increases in this population, so does the population of women with a history of prior cesarean birth having to choose a method of delivery for their next delivery. For women who are well-suited for it, attempting a vaginal birth after a cesarean is a safe and evidence-supported option. Estimates suggest that 60%–80% of these women would successfully have a vaginal delivery if they choose to try (4). In 2019, a study found that in the United States rates of trial of labor after a cesarean (TOLAC) have increased from 14.4% in 2010 to 19.6% (5). However, a systematic review found that in Latin America (Argentina, Brazil and Chile) there was a higher preference for a repeated cesarean in comparison to high income countries (6). In Guatemala, women with a history of a prior cesarean who choose elective repeat cesarean birth account for the largest proportion of the overall cesarean birth rate (7, 8).

Prior research has found that increased maternal age, education, information given to patients after the prior cesarean birth, the lack of privacy and the delivery of high-quality respectful care have been associated with a repeat cesarean birth (9, 10). Based on our previous research in Guatemala, reduced parity, delivering at a facility (as compared to home), and delivering by a physician were found to be associated with repeat cesarean births (11). While there are clear data from high-income countries on the risks and benefits of elective repeat cesarean delivery (ERCD) compared to trial of labor after cesarean section, the decision about method of delivery is still very personal in terms of what risks women are willing to assume for themselves and their babies. Many communities have turned to decision tools to help them interact with women on making this decision (12–20). There are also some data about when in pregnancy it is best to have these conversations (21). However, there is limited information about women's general perspective regarding decision making in mode of delivery in Latin America (22).

Little research has explored the needs and values of this Guatemalan population with respect to accessing a decision aid for deciding on mode of birth after cesarean. Therefore, what is available does not meet women's needs, limits women's ability to understand their choice on method of delivery, as well as the physician's ability to counsel patients. To fill this gap, we explored the views and opinions of women regarding a decision aid for women with a history of prior cesarean birth as well as the perspective of the providers for this community. Specifically, we identified factors to help shape the decision aid content and method of delivery within the hospital setting. We intended for this study to provide context for any future decision aids that might be developed in this region to counsel patients on mode of birth after a prior cesarean birth. This information is vital not only for shaping future clinical practice guidelines and decision-making processes related to the mode of delivery during pregnancy but also for guiding the broader implementation of shared decision-making in Latin America to address other women's health issues.

## Methods

We conducted a qualitative content analysis study using in-depth semi-structured interviews. These interviews examined the perceptions of women who had previously undergone a cesarean section, as well as those of resident and attending obstetrician-gynecologists, regarding delivery methods in subsequent pregnancies complicated by a prior cesarean.

### Setting

In the Southwest region of Guatemala, the Trifinio is located at the intersection of three coastal lowland departments: San Marcos, Retalhuleu, and Quetzaltenango. This region exports primarily bananas and palm oil owned by substantial agribusiness enterprises. The more than 20 small communities that make up the Trifinio have an impoverished rural population of 30,000 (23). These communities lack access to health services, education, and reliable clean water. A partnership between the University of Colorado and local agricultural businesses in the lowlands of Southwestern Guatemala resulted in the creation of the Center for Human Development (CHD) in 2011 (24). The CHD operates a family clinic, dental clinic, clinical laboratory, pharmacy and community-based maternal, child, and adolescent health programs. Madres Sanas is a maternal health program which includes a community-based home visitation service delivered by specially trained nurses during the prenatal and postnatal periods (24). This program includes regular prenatal visits, postpartum visits, and additional unscheduled visits as needed. The nurses also provide education on topics such as danger signs of pregnancy, nutrition, breastfeeding, and contraceptive use. Participants in this program are typically multiparous, married or living with a partner, have received elementary school education, and are not employed (11).

### Study participants

Between November 2019 and February 2020, eligible participants were recruited for this qualitative study. The women who participated in our study were recruited from community outreach programs within the CHD. These programs provide maternal and child health to pregnant women and children in the surrounding area. The study coordinator, the nursing supervisor of the Madres Sanas program (24), approached women who recently delivered at their 40-day postpartum visit by cesarean birth and invited them to participate. Interviews with women took place in a consultation room at the CHD. To obtain the convenience sample, nurses who visited women for their postpartum visits offered participation in the study. Obstetricians (residents and attendings) engaged in clinical care at the Coatepeque Hospital in Coatepeque were recruited and were invited to participate in our study.

### Data collection

All participants were consented and interviewed by the study team in a private conference room. Our interview guide was designed using the socioecological model which we used for analysis (25). Prior to analysis, we divided our codebook into the needs and values of this population with respect to accessing a decision aid for birth after cesarean. Based on the sociological framework developed in designing our own study, we placed a special emphasis on social considerations in the codebook (26).

The interview guide for women focused on understanding their ideal subsequent birth and their attitudes and beliefs about mode of delivery after a prior cesarean birth using the same framework. The interview guides for the obstetricians focused on knowledge, attitudes, and practices related to mode of delivery for women with a history of prior cesarean birth, including clinical indications and social considerations. Furthermore, questions related to the opinions and perspective regarding a decision aid tool were asked of women and providers. These questions focused on what type of information women would like to receive after a cesarean birth, how the information should be delivered in that context, and who is the best person to deliver the information. The interview guides were not revised over the course of the study. All interviews lasted between 15 and 45 min and were audio recorded. A native Spanish speaker took detailed interview notes during the interviews. We aimed to recruit 20–30 participants, as this number would allow us to reach saturation of relevant themes.

### Data analysis

A HIPAA-certified professional transcriptionist in the language of the interview transcribed the Spanish audio recordings verbatim. The transcripts were then professionally translated into English. When the data were prepared, they were sent securely to the senior professional research assistant who stored the data on password-protected servers. All translated transcripts were validated by the interviewer, who listened to the audio file and verified both the transcription and translation for accuracy. Validated transcripts were reviewed for integrity and uploaded into ATLAS.ti (ATLAS.ti Scientific Software Development, Berlin, Germany) in preparation for analysis in a de-identified format, with interviews saved as a combination of numbers and letters, allowing for anonymization of the content. The codebook was then applied to all transcripts by the members of the research team. Two members of the research team (AJ-Z and MA) read the same two transcripts and through consensus agreed upon additional inductive codes. If consensus could not be reached, MSH read the same two transcripts to facilitate discussion and achieve consensus. To establish coding standards, sections of a third transcript were double-coded to assess intercoder reliability. When the codebook was finalized, the remaining transcripts were coded by the same two members of the research team. Next, the coded data was analyzed within and between participant types (women and physicians) to identify the major themes and illustrative quotes that captured the participants’ perspectives (27). Study participants did not provide feedback on the findings.

## Results

All the interviews were conducted in February 2020 in Spanish by a native speaker. We conducted a total of *n* = 30 interviews with physicians (*n* = 10) and with women who had a prior cesarean birth (*n* = 20). Study participant characteristics are presented in Table 1, only participants with complete demographic data were included. Three recurrent themes related to decision aid implementation were identified through the analysis. Verbatim quotations are used to illustrate themes as well as a range of views expressed in the interviews. Qualitative analysis of interviews of women and providers indicated key themes that emerged from the data: (1) decision aids will be a helpful tool, (2) content of the decision aid should include culturally tailored risk and benefit information, and (3) trusted providers should share and facilitate the decision aid discussion.

**Table 1:** Demographic characteristics of the study participants.

	Women (*n* = 20)	Providers (*n* = 8)^a^
Age, yr		
Mean (Standard Deviation)	23.03 (5.8)	35.44 (8.6)
Marital Status, *n*(%)		
Single/Divorced/Separated/Widowed	18 (90.0%)	4 (50.0%)
Married/living with partner	2 (10.0%)	4 (50.0%)
Education, *n*(%)		
No formal education	1 (5.0%)	
Primary School (1–6)	13 (65.0%)	
Secondary School (7–10)	3 (15.0%)	
Diversified Secondary (+11)	1 (5.0%)	
University	2 (10.0%)	
Parity		
1	9 (45.0%)	
2	8 (40.0%)	
3	3 (15.0%)	
Gender (Female)	20 (100%)	4 (50.0%)
Year of graduation of Medical School		
2006		1 (12.5%)
2014		1 (12.5%)
2015		2 (25.0%)
2016		1 (12.5%)
2018		2 (25.0%)
2019		1 (12.5%)

^a^ Table of participants with non-missing data.

### Practicality of decision aids

In general, all participants spoke positively about the concept of decision aids and considered that having a decision aid for women with a prior cesarean birth would be useful not only for women but also for physicians. One said, “Yes, it would help them a lot because they think that if they had a C-section, I can’t have vaginal delivery.” A different physician shared a similar opinion: “The truth is that it would help a lot, because there are many risk factors, not only economic, social, environmental, family.” Another one said, “That would be a useful tool.” Women shared similar opinions. One women reported, “Yes, I would like to know more– more information on what a c-section can give [offer] you, [in comparison] of a normal delivery. It would be great to learn more.” Similarly, another participant said “Oh, well yes, [a decision aid will] give me the information to see what [options] I can do later.”

### Decision aid content

Participants agreed that the content of the decision aid tool should include benefits and risks for women and baby. One mother shared, “*I think that every woman should be informed about how the baby is coming, if the position is appropriate to have a vaginal delivery; if it's not sideways or in a breech position because if the baby is in that position, you will need a C-section delivery. You should also know if the baby's weight is correct because sometimes when they are too small many complications could arise during vaginal delivery. It would also be helpful to know if the baby's heart rate is normal because when you have a vaginal delivery the baby's heart rate can speed up or slow down. I think that information is crucial to know.”*

Similarly, a physician emphasized the importance of including this information, “*because the [patients] think that if they had a C-section, can’t have vaginal delivery. But if they are told the advantages of a vaginal delivery, the faster healing, the risk of infection is less, that is, all that…the patients would understand and maybe they would like vaginal births more.”* Another physician added, “*[the decision aid should] explain what are the benefits and what are the complications that could also have and what are also the benefits of a vaginal delivery, which will have a faster recovery period, you will not be exposed to anesthesia, you will not have problems with something else that could happen*.”

An additional important topic was related to their preference of how the content should be displayed. Participants were aware that the decision aid tool should have more graphic content than written material due to the literacy limitations of the population. One physician explained, “With figures, so they understand better, because we explain them in a way, in their language so that they can understand better, but it would be quite supportive to have a visual material…So, where there would be images of the uterus, the scar, the incisions that are made.” A women explained, *“what a C-section delivery is like and what a normal one is like, I have it all the pictures here… because there are some moms who have not yet finished their education, that have no education. And in reality, it's difficult for them to get help with letters, they can’t read or can’t write”*.

During our interviews, we also explored the influence of women's families on their decisions regarding spacing and delivery. One women reported, “my husband and I said that we wanted to have the baby at home, but as I had complications, I was unable to have the baby at home. So, he said, ‘We’d better go because the baby—I don’t want you or the baby to die because there's a risk that you could die, so what's the point of having you and not my baby.’ So, that's why [my husband] took me to where I had the C-section.” Other women share a similar experience, “Yes, they were sending me back home but my husband said no, to stay there until they took me in. And I had to stay. And that's when the doctor said that I needed to have a C-Section.”

Physicians also reported seeing this dynamic with their patients. One said, “*for example, I explain something to her [the woman], but the mother-in-law explains otherwise, the neighbor explains something else, the husband explains something else. So, sometimes what we deal with is, she came with her husband or mother-in-law, or with her mother, and we can explain: ‘Look— maybe that would be another person's opinion, because that is what happens most of the time*.” One explains, “and then, not anymore, because the mother-in-law decided otherwise.” Another physician responded, “many patients who say: ‘I don't want to have surgery because my husband does not want to,’ or ‘my husband [will] beat me,’ or ‘my husband this.’ So, there are some patients who do want [surgery], but because of the fear of her husband they do not [have it]. Therefore, I think [using the decision aid] as a couple, not only the mother, but as a couple, so that this can also come and make the husband aware”.

### Facilitation to implementation of decision aids

Most participants agreed that a trusted provider from the healthcare system should facilitate decision aid discussion. Most physicians reported that the hospital personnel were the most qualified to implement the decision aid tool. One physician shared, “I think all the staff [doctors and nurses], I think it's all the staff that is involved in this situation.” Another physician shared a similar opinion, “I think that it is not only the doctor's responsibility, but something that can be shared with all the staff, all the staff who has contact with the pregnant women should know what are their advantages, their disadvantages, why yes, why not, and what can we offer.”

Women also share similar opinions. One women said, “It would be with a doctor or a nurse if that were the case.” Another participant reported, “[I will want] a doctor.” However, among women, some shared that they would also like the community traditional birth attendant (comadronas) to also be involved in the decision. One explained “Maybe the traditional birth attendant can have that information…But a doctor would be better.” Another one said she “[would like to discuss decision aid] with a traditional birth attendant.”

## Discussion

The topic of using a decision-making support tool for the mode of delivery after a previous C-section remains largely unexplored in Latin America. Our study highlights several critical aspects regarding the development and implementation of a decision aid tool for women considering a trial of labor after a C-section (TOLAC). Both physicians and women who participated in our study expressed strong support for the concept of a decision-aid tool. They acknowledged its potential to facilitate informed decision-making by clearly presenting the risks and benefits associated with different delivery options, a perspective supported by previous literature (28, 29). One key insight from our findings is the consensus on the need to address not only the medical aspect of this type of birthing decision, but the risks and benefits of this decision-making process.

Physicians and women alike emphasized the importance of incorporating content that is comprehensible to a broad audience, including those with low literacy. The inclusion of visual aids such as graphs and figures, alongside clear written content, emerged as crucial for enhancing understanding. This aligns with existing literature that underscores the necessity of tailoring decision aids to meet the diverse needs of the target population (30, 31). Furthermore, the shared responsibility of decision-making between the patient and the family unit was a notable theme. Participants agreed that the decision aid should not only inform the woman but also engage her family members, reflecting a more holistic approach to decision-making, similar to the strategies explored in decision aid tools for hospice care (32).

This finding underscores the need for the tool to be adaptable to different family dynamics and to provide guidance that can be discussed and deliberated upon within the family context. Physicians in our study were unanimous in their opinion that the presentation of the decision aid should be managed by a healthcare professional, such as a provider or nurse. This is likely due to the need for expert guidance to address questions and provide personalized support. Women in our study supported this viewpoint but also suggested that traditional birth attendants, who are trusted community figures, could play a valuable role in disseminating the information. This suggestion points to the importance of integrating community-based perspectives into the development and implementation of decision aids, particularly in settings where traditional birth attendants have significant influence.

Our findings build on previous research by extending the discussion to the specific context of TOLAC and the unique considerations it entails. While previous studies have highlighted the general acceptance and effectiveness of decision aids in similar contexts (13, 33), our research introduces a novel perspective on the necessity of including family dynamics and community-based figures in the decision-making process. This approach not only enhances the relevance of the decision aid but also acknowledges the social and cultural dimensions of childbirth. To our knowledge, this is the first paper that highlights the importance of tailoring a decision aid tool for trial of labor after a C-section focusing on a shared decision-making process between the women and the entire family unit. In addition, participants also suggested the inclusion of traditional birth attendants as trusted community members capable of the dissemination of a decision aid tool.

### Limitations

Despite the significant insights gained from these findings, several limitations must be considered. First, one limitation is our study was limited by the convenience sampling of our populations both in the hospital and in the community setting. Therefore, the findings, however informative, may not be representative of the experiences of those participants declined to participate, those who were not a part of the Madres Sanas program or those providers who declined participation. Thus, the findings may not be generalizable to the entire rural Guatemalan population.

Second, even when the lead interviewers were a native and fluent Spanish speaker, their status as outsiders may have influenced the responses of women in the Trifinio as well as physicians in the hospital. Future research should examine the perspectives of rural women who have previously had a C-section by recruiting women who either did not engage with a maternal health program or those who dropped out. Additionally, to gain a comprehensive understanding of providers’ perspectives, medical students as well as support staff (nurses and medical assistants) should be considered. This approach will provide a holistic view of all medical personnel involved with patients delivering children after a previous C-section. Although we believe that a decision aid would be well received and integrated into standard care, it remains essential to create an implementation module. We need to observe its association with maternal birth planning, including the desired method of delivery, the ultimate method of delivery, location of delivery, and neonatal intensive care unit admission as primary health outcomes, and compare these with historical data to assess effectiveness and efficacy.

## Conclusions

Decision aid tools around mode of birth are needed to narrow the birthing education gap for women with a prior cesarean. Such decision aids on the options for the mode of birth after a cesarean should be based on what is medically available and women's personal experiences. Findings from this study suggest that decision aids might be well accepted among this population and might be useful as long as their content is tailored to participant's literacy level and target the entire family unit. These findings highlight the importance of developing an inclusive and innovative approach to patient messaging and education around mode of delivery after a prior cesarean. This approach could improve the quality and dissemination of information given to support women and providers regarding mode of birth in the facility setting. Finally, the implementation of evidence-based practices around natural labor after cesarean could potentially assist in further improving the quality of care at Coatepeque Hospital.

## Data Availability

The raw data supporting the conclusions of this article will be made available by the authors, without undue reservation.
